# Boosting photoresponse in silicon metal-semiconductor-metal photodetector using semiconducting quantum dots

**DOI:** 10.1038/srep37857

**Published:** 2016-11-25

**Authors:** Chandan Biswas, Yonghwan Kim, Young Hee Lee

**Affiliations:** 1 Center for Integrated Nanostructure Physics, Institute for Basic Science (IBS), Suwon 16419, Republic of Korea; 2 Department of Energy Science, Sungkyunkwan University, Suwon, 16419, Republic of Korea; 3 Electrical and Computer Engineering, University of Texas at El Paso, El Paso, TX 79968, USA

## Abstract

Silicon based metal-semiconductor-metal (MSM) photodetectors have faster photogeneration and carrier collection across the metal-semiconductor Schottky contacts, and CMOS integratibility compared to conventional p-n junction photodetectors. However, its operations are limited by low photogeneration, inefficient carrier-separation, and low mobility. Here, we show a simple and highly effective approach for boosting Si MSM photodetector efficiency by uniformly decorating semiconducting CdSe quantum dots on Si channel (Si-QD). Significantly higher photocurrent on/off ratio was achieved up to over 500 compared to conventional Si MSM photodetector (on/off ratio ~5) by increasing photogeneration and improving carrier separation. Furthermore, a substrate-biasing technique invoked wide range of tunable photocurrent on/off ratio in Si-QD photodetector (ranging from 2.7 to 562) by applying suitable combinations of source-drain and substrate biasing conditions. Strong photogeneration and carrier separation were achieved by employing Stark effect into the Si-QD hybrid system. These results highlight a promising method for enhancing Si MSM photodetector efficiency more than 100 times and simultaneously compatible with current silicon technologies.

Metal–semiconductor–metal (MSM) photodetectors are under intensive research focus due to faster light detection capabilities and higher bandwidths^1^. Faster photogeneration and carrier collection across the metal-semiconductor Schottky contact result in fast photocurrent compared to conventional p-n junction photodetectors^2^. Different types of semiconductors such as GaAs, InGaAs, TiO_2_, Single walled carbon nanotubes, and graphene have been investigated as the semiconducting component in MSM photodetectors^3^,^4^,^5^,^6^,^7^. However, silicon-based photodetectors are among the most attractive materials due to its monolithic integratibility with complementary metal oxide semiconductor technologies^8^,^9^,^10^,^11^. Simultaneously strong spatial confinement of electronic wave functions enhances carrier-carrier interaction in semiconducting quantum dot (QD). This leads to numerous exotic electronic and optoelectronic properties in QDs, significantly different from their bulk states^12^,^13^,^14^,^15^. Exciton (or carrier) multiplication^13^, Stark effect^14^, and band shifting^15^ are among the key optoelectronic properties of semiconducting QDs that could be exploited to improve MSM photodetector operations. Therefore, a highly efficient and monolithic MSM photodetector device can be designed by incorporating semiconducting QDs in Si-based MSM device structure (Si-QD). In this article, we demonstrate the enhancement strategies of Si MSM photodetector by incorporating an uniform coating of CdSe QDs on Si surface. Photocurrent on/off ratio was significantly boosted from 5.2 (in Si MSM photodetector) to 562 in Si-QD MSM photodetector devices. This simple QD incorporation method significantly increased photocurrent on/off ratio up to nearly 108 times in Si-QD MSM photodetector compared to conventional Si devices. Moreover, photocurrent on/off ratio can be tuned using applied source-drain and substrate biasing (V_sub_) techniques in Si-QD MSM photodetector. Tunable photodetector on/off ratio was demonstrated from 2.7 to 562 by applying source-drain and substrate biasing combinations. This tunability can be highly useful for external load-matching photodetector applications. The discrete energy levels and strong Coulomb interaction in QDs result in a sharp increase in photogeneration and consequent improvements in Si-QD photodetectors. Furthermore, device biasing technique invoked supplementary enhancement in photogenerated charge carrier separation and improved Si-QD device performance. Strong photogeneration from Si-QD hybrid structure and consequent shift in the transition energies due to device biasing was verified by Stark effect overserved from photoluminescence investigations. These results suggest strong photodetector application opportunity compatible with conventional silicon based complementary metal-oxide semiconductor technology.

## Results and Discussion

**Figure 1:**
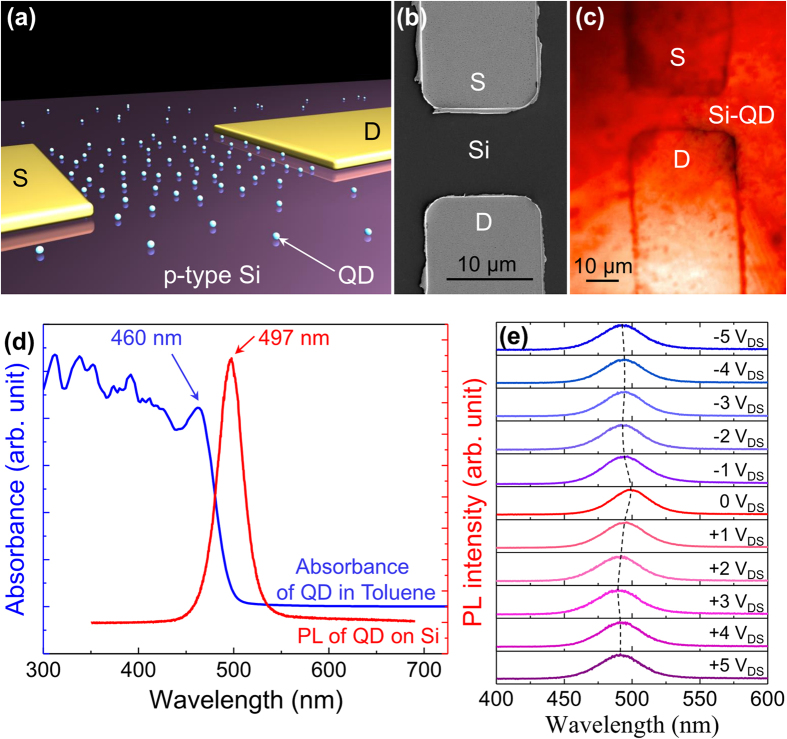
(**a**) Schematic model of CdSe decorated Si MSM device consists of source (S) and drain (D) Cr/Au (5 nm/50 nm) metal electrodes. (**b**) SEM micrograph of Si MSM device without QDs. Si devices were fabricated with 10 μm channel length and various different channel widths (not shown in the figure). (**c**) Photoluminescence intensity plot (at 497 nm) of the QD decorated Si MSM device with a 355 nm diode-pumped solid state (DPSS) laser excitation. (**d**) Absorbance (QD dispersed in toluene solution) and PL (QD deposited on Si substrate) spectrum of CdSe QDs. (**e**) PL peak shift under different substrate bias.

Si MSM photodetectors were fabricated by thermal deposition of metal electrodes on top of a p-type Si substrate. Two source-drain Cr/Au (5 nm/50 nm) metal electrodes were deposited with a 10 μm channel length and different channel widths. Figure 1(a) represents the schematic model of CdSe QDs decorated on top of the Si MSM device. Uniform dispersion of CdSe QDs (average diameter: 2.2 nm) in toluene solution was spin-coated on top of the Si MSM device in order to achieve uniform QD distribution. The spin-coating condition was optimized and kept constant to 3500 rpm during one minute throughout the experiment. Figure 1(b) and (c) demonstrate Si and Si-QD device morphologies before and after QD decoration, respectively. Field emission scanning electron micrograph (FE-SEM) shown in Fig. 1(b) of Si MSM photodetector device before QD decoration. Uniform QD deposition on Si MSM device could be verified by the continuous photoluminescence (PL) intensity plot shown in Fig. 1(c). A QDs deposited PL peak was obtained at 497 nm, which was compared with the absorption edge (460 nm) of the QDs dispersed in toluene as demonstrated in Fig. 1(d). The pristine QDs dispersed in toluene resulted in sharp absorption in the wavelength ranging from near UV to 460 nm. When these QDs are deposited on Si substrate, they triggered strong PL peak around 497 nm. The PL peak position is the same as the absorption edge, as expected. The PL transition peaks were compared with varied source-drain voltages (V_DS_ from +5 V to −5 V) in order to investigate the electric field induced photoactivity in Si-QD MSM device structure. Figure 1(e) demonstrates a sequential blue shift in the PL peak under +5 V (PL peak at 491.3 nm) and −5 V (peak at 492.7 nm) applied V_DS_. The discrete energy levels of quantum dots (confined in low dimension) and their transition energy shift due to the Stark effect under external electric field were observed previously, causing these PL peak shifts^14^,^16^. An asymmetric change of electron and hole wave functions and the electron-hole overlap integral under an external electric field in a quantum confined structure result in Stark effect^14^,^16^. These results highlight that the radiative recombination transition energies (PL peak shift) in QDs can be tuned by applied substrate potentials.

**Figure 2:**
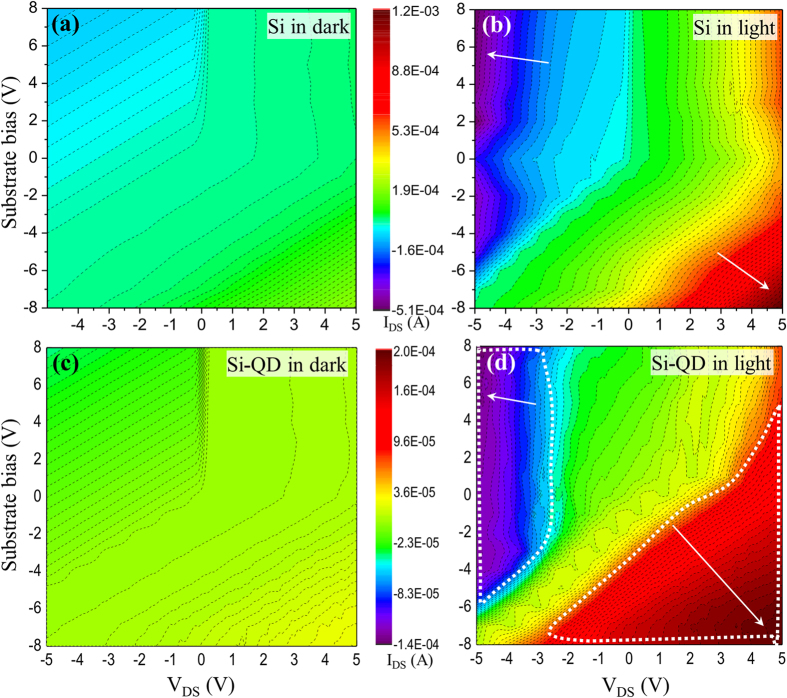
(**a**,**b**) I_DS_ current mapping plot of Si device without QDs using different source-drain bias (V_DS_) and substrate biasing conditions under dark (**a**) and in light (**b**). (**c**,**d**) I_DS_ color mapping plots of Si-QD device with identical V_DS_ and substrate biasing conditions under dark (**c**) and in light (**d**) conditions. Dashed lines showed high current region in (**d**).

Electrical current measurements were performed in order to compare photodetector efficiencies in dark and under light conditions. A standard AM 1.5 light source (100 mW power) was used to illuminate samples during the current measurements. The source-drain current (I_DS_) was collected between the source and drain electrodes under variable applied V_DS_ potentials. In addition to that, I_DS_ was further measured under applied bias between the source and Si substrate (substrate bias, V_sub_) to determine the variations in photoactivity. Figure 2 compares I_DS_ current mapping of Si and Si-QD devices under dark and light conditions. Figure 2(a) and (b) were plotted with identical XYZ axes (X: V_DS_ = ±5 V, Y: V_sub_ = ±8 V, and Z: I_DS_ from 1.2 mA to −0.51 mA) in order to compare Si MSM photodetector under the dark and illumination conditions. The Si MSM photodetector in dark showed low current level in the range from 0.19 mA to −0.12 mA (Fig. 2(a)). The device exhibited high current particularly at high bias regions (see arrows in the red and violet regions in Fig. 2(b)) under AM 1.5 light illumination. Similar investigations were performed in Si-QD MSM photodetectors and represented in Fig. 2(c) and (d) respectively. High current region in Si-QD samples was significantly larger than Si device as highlighted by the dotted lines in red and violet regions shown in Fig. 2(d). Photoexcited charge carriers generated from the CdSe QDs could be easily separated by biased Si substrate at a lower potential compare to Si device without QDs^17^. The enhancement in the photocurrent due to the incorporation of QDs was further evaluated and discussed later.

**Figure 3:**
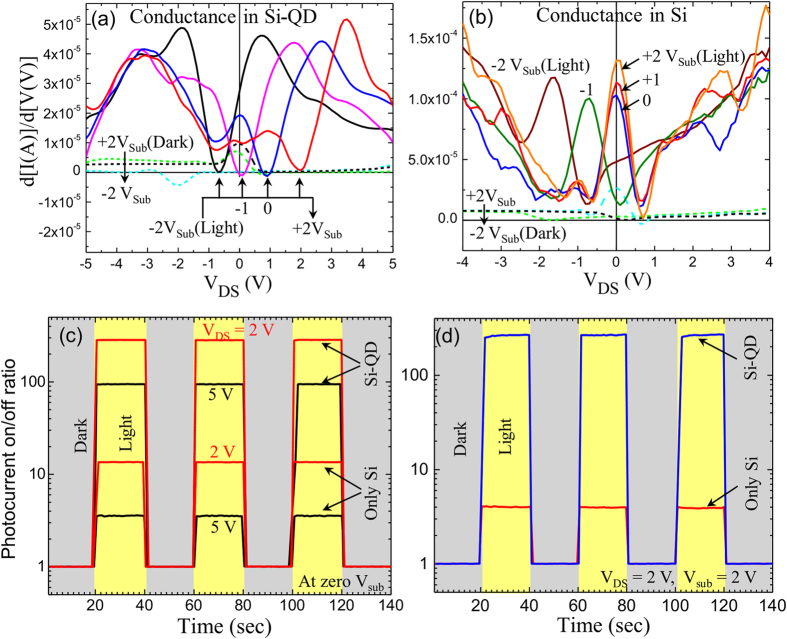
(**a**,**b**) Device conductance (in dark) and photoconductance (under light) comparisons for the devices with QDs (**a**) and without QDs (**b**) under different V_DS_ biasing conditions. (**c**) Photocurrent on-off ratio (ratio between the dark current and current under light) for the Si and Si-QD devices without substrate bias as the light was turned on & off. (**d**) Photocurrent on/off ratio for the Si and Si-QD devices with a fixed source-drain and substrate bias (+2 V).

Dark conductance (device conductance in dark) and maximum photoconductance (conductance under light) were observed in the Si-QD devices (Fig. 3(a)) in the order of 5 μS and 50 μS range, respectively. Here a photoconductance trough was observed which shifted towards higher positive V_DS_ values with increasing V_sub_ conditions and could be related to the carrier polarity switching during opposite biasing conditions^11^. The dark conductance (~5 μS) was observed 20 times smaller than the photoconductance (~100 μS) of the Si device as shown in Fig. 3(b). A photoconductance peak was observed near low V_DS_ region due to low photogenerated carrier scattering^11^. The photoconductance peak was shifted towards negative V_DS_ biasing under negative V_sub_ conditions (see Fig. 3(b)). The photocurrent of the Si and Si-QD devices was compared with and without substrate biasing condition in Fig. 3(c) and (d) respectively. Figure 3(c) represents the photocurrent on/off ratio of the Si and Si-QD devices with zero V_sub_ and constant V_DS_ biasing conditions. The photocurrent on/off ratio in the Si-QD device was near about 25 times higher than that of Si device both under +2 V and +5 V V_DS_ biasing conditions. These results suggest a photocurrent enhancement in the Si-QD device due to the CdSe QDs incorporation on the Si surface. Furthermore, the photocurrent on/off ratio can be further increased by applying substrate bias in Si and Si-QD devices. The photocurrent on/off ratio was enhanced up to 265 in Si-QD device under +2 V substrate and V_DS_ biasing condition (see Fig. 3(d)). These results highlight a significant increase in the net current due to the addition of significantly high photogenerated current and further enhancements in the photocurrent on/off ratio in Si-QD MSM device structure.

**Figure 4:**
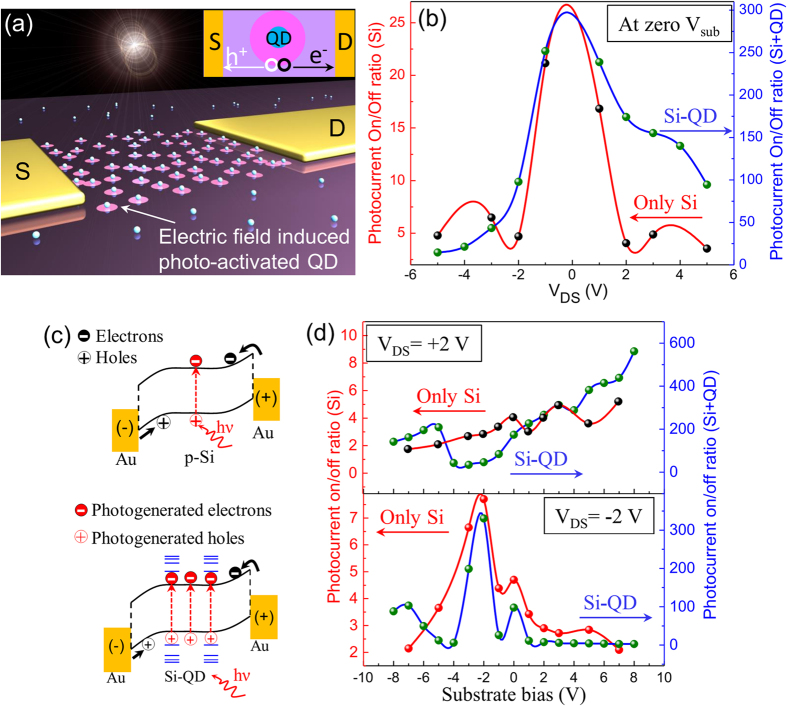
(**a**) Schematic representation of Si-QD device decorated with electric field induced photoactive QDs regions (shown in violet circular area) in source-drain channel. Inset represents the schematic model of photocarrier generation and separation. (**b**) Variation in photocurrent on/off ratio of Si and Si-QD device against variable applied V_DS_ without substrate biasing (V_sub_). (**c**) Schematic model of the Si and Si-QD band alignments under illumination. (**d**) Variation in photocurrent on/off ratio of Si and Si-QD device with variable applied V_sub_ at the same V_DS_ potential (2 V) with different polarities.

The photodetector output on/off ratio can be controlled with a combination of V_DS_ and V_sub_ as shown in Fig. 4. Figure 4(a) illustrates the schematic model of the Si-QD device with electric field induced photo-activated QD regions. Photogenerated electron and hole carriers can be separated from these regions under applied V_DS_ potentials (inset). The photocurrent on/off ratio dependences on V_DS_ was compared for the Si and Si-QD devices in Fig. 4(b). Low V_DS_ potentials resulted in low kinetic charge carriers and comparably low carrier scattering in the Si and in Si-QD devices and resulted in a sharp photocurrent on/off ratio peak near low V_DS_ bias. However, significantly high photocurrent on/off ratio was observed in the Si-QD device (~250 at −1 V V_DS_) compare to Si device (~20 at −1 V V_DS_) due to the photogeneration from the CdSe QDs. This gave us a unique capability to control photodetector on/off ratio by using externally applied source-drain and substrate potentials. Variations in applied substrate bias were also a crucial factor to tune photocurrent on-off ratios by controlling photogenerated carrier separations. Figure 4(c) represents the schematic diagram of the Si and Si-QD band alignments in which photogenerated charge carrier generation in Si-QD was compared with Si device. The pristine Si MSM device resulted in moderate photocurrent (see Fig. 2). The photocurrent on/off ratio up to 13.5 was observed under 2 V V_DS_ conditions (see Fig. 3c). On the other hand, significant increments in the photocurrent (Fig. 2d) and the corresponding photocurrent on/off ratio of around 282 (shown in Fig. 3c) were obtained in Si-QD MSM devices due to QD-induced photogeneration. This could be due to the photogenerated charge carrier generation from strong Coulomb interaction in quantum dots (confined in low dimension) under photon excitation^17^,^18^,^19^. The number of free charge carrier contributed towards the net current flow (see Fig. 4c) in the MSM device was significantly increased due to the photogeneration from QDs in Si-QD structure compared to Si. This was demonstrated by the device schematics in Fig. 4c in which photogenerated charge carriers (red color) significantly increases in Si-QD compared to Si device. Figure 4(d) represents photocurrent on/off ratio dependence at V_DS_ = ± 2 V and different V_sub_ conditions. The photocurrent on/off ratio was increased with increasing substrate bias both in the Si and Si-QD devices. The maximum photocurrent on/off ratio at V_sub_ = 8 V was observed near to 5.2 in Si device and 562 in Si-QD device (~108 times higher). However, the minimum photocurrent on/off ratio in Si-QD devices was observed nearly around 2.7 (close to the Si values) at V_sub_ = + 8 V and V_DS_ = −2 V biasing conditions. On the contrary, positive V_DS_ potential (+2 V) under identical substrate bias (+8 V) conditions resulted in very high photocurrent on/off ratio up to nearly 562 (see Fig. 4(d)). The photocurrent on/off ratio in this case was nearly 208 times higher than the minimum value (2.7) and could be tuned to any intermediate values by applying suitable combinations of V_DS_ and V_sub_ biasing conditions. Therefore, 108 times increment in the photogeneration and 208 times photocurrent on/off ratio tunability was controlled by applying source-drain and substrate biasing combinations. The enhanced photogenerated carrier separation induced by electric field induced photo-activated QDs played crucial role behind the photocurrent on/off ratio enhancements and tuning abilities in Si-QD MSM device structure.

In summary, an enhancement strategy of Si MSM photodetector was demonstrated by incorporating CdSe QDs uniformly coated on top of the Si surface. More than 108 times higher photocurrent on/off ratio was achieved in Si-QD MSM devices (on/off ratio ~562) compared to Si devices (around 5.2). Moreover, a wide range of tunable photocurrent on/off ratio in Si-QD photodetector was demonstrated (from 2.7 to 562) by applying suitable combinations of source-drain and substrate biasing conditions. This could be highly applicable for the photodetector applications where tunable photodetector efficiency is required. The discrete energy levels and strong Coulomb interaction in quantum dots (confined in low dimension) resulted in a sharp increase in photodetector efficiencies. Moreover, the substrate-biasing technique invoked further enhancement in photogenerated charge carrier separation in QDs and improves Si-QD device performance. 108 times increment in the photogeneration and 208 times photocurrent on/off ratio tunability were demonstrated by applying source-drain and substrate biasing combinations. This method is compatible with the current silicon technologies and the obtained results highlight significant enhancement opportunity in the Si MSM photodetector efficiency up to more than 100 times.

## Materials and Methods

Si MSM photodetectors were fabricated by thermal deposition of metal electrodes on top of a p-type Si substrate. Two source-drain Cr/Au (5 nm/50 nm) metal electrodes were deposited with a 10 μm channel length and various different channel widths. Uniformly dispersed CdSe QDs (average diameter: 2.2 nm) in toluene solution was spin-coated (3500 rpm for one minute) on top of the Si MSM device in order to achieve uniform QD decoration and fabricate Si-QD MSM photodetector structures. Electrical performance of the devices were measured using Keithley 4200 semiconductor analyzer in dark and light conditions (using AM 1.5 light source with 100 mW optical power).

## Additional Information

**How to cite this article**: Biswas, C. *et al*. Boosting photoresponse in silicon metal-semiconductor-metal photodetector using semiconducting quantum dots. *Sci. Rep.* **6**, 37857; doi: 10.1038/srep37857 (2016).

**Publisher's note:** Springer Nature remains neutral with regard to jurisdictional claims in published maps and institutional affiliations.
